# Systematic hybrid laparoscopic and endovascular treatment of median arcuate ligament syndrome: A single-center experience

**DOI:** 10.3389/fsurg.2023.1169681

**Published:** 2023-04-19

**Authors:** Michael Schneider, Justine Longchamp, Emilie Uldry, Jean-Marc Corpataux, Amaniel Kefleyesus, Nermin Halkic

**Affiliations:** ^1^ Department of Visceral Surgery, Lausanne University Hospital and University of Lausanne, Lausanne, Switzerland; ^2^ Department of Vascular Surgery, Lausanne University Hospital and University of Lausanne, Lausanne, Switzerland

**Keywords:** median arcuate ligament syndrome, celiac trunk compression, Dunbar syndrome, endovascular treatment, minimally invasive surgery, laparoscopic surgery

## Abstract

**Background:**

Median arcuate ligament syndrome (MALS) is caused by celiac trunk (CT) compression by the median arcuate ligament. Clinically, this pathology varies from postprandial pain (Dunbar syndrome) to a life-threatening hemorrhage because of a rupture of a gastroduodenal artery aneurysm (GAA). Due to the low prevalence of this disease, there is no standard management for MALS.

**Material and method:**

This was a single-center, retrospective study of 13 patients. Two groups were identified on the basis of the initial clinical presentation: those operated for a GAA rupture (bleeding group—BG) and those operated electively for Dunbar syndrome (Dunbar syndrome group—DG). The primary endpoint was 30-day postoperative complications of a systematic laparoscopic release of the median arcuate ligament and stenting during the same procedure.

**Results:**

Seven patients (54%) underwent elective surgery. Six patients (46%) underwent semiurgent repair under elective conditions post-embolization for GAA bleeding. The total operative time was longer in the BG (*p* = 0.06). Two patients in the BG suffered early major complications and needed reintervention, and those in the DG had a lower comprehensive complication index. No mortality was reported at 30 days. Overall median length of stay was 5 days (IQR: 3.5–15.3). Patients in the DG had a significantly shorter length of stay (*p* = 0.02). At 6 months, the primary and secondary CT stent patencies were 82% and 100%, respectively. There were no high-flow GAA recurrences.

**Conclusions:**

A combined approach of laparoscopic release of the median arcuate ligament and stenting during the same procedure is feasible and safe, and this approach must be systematically discussed in symptomatic patients.

## Introduction

Median arcuate ligament syndrome (MALS) is the result of a compression of the celiac trunk (CT) by the median arcuate ligament (MAL), which leaves fibrous attachments on the diaphragmatic crura. Anatomically, the prevalence of MALS is up to 24%; however, only 1% of these patients are symptomatic (1). Clinical manifestations such as diffuse pain, nausea, vomiting, weight loss, or food fear, are nonspecific. This diagnosis of exclusion is also known as Dunbar syndrome (DS), which often leads patients to undergo several investigations with abdominal computed tomography (CT-scan), ultrasonography, or endoscopy. Duplex inspiration–expiration ultrasonography or CT-scan angiography can also help to confirm the diagnosis. In late stages, patients may present with a potentially fatal hemorrhage due to a rupture of a gastroduodenal artery aneurysm (GAA). The pathophysiology of late-stage MALS GAA has not yet been fully understood and descriptions in the literature are scarce, with mainly small series and case reports (2–5).

It has been hypothesized that increased blood flow in the superior mesenteric artery (SMA) secondary to CT compression induces high-flow GAA, generally in the inferior pancreatico-duodenal artery (IPDA). Due to the rarity of this disease, treatment is not well established, and there is currently no gold standard of care.

We performed a retrospective single-center study including all patients who underwent MAL release during the past 10 years at our institution. We proposed a hybrid procedure by a laparoscopic release of the MAL with angioplasty and stenting of the CT.

## Methods

In this study, the medical records of all patients who underwent MAL release between January 2011 and December 2021 were reviewed. The inclusion criterion for the study was patients >18 years old. Data were retrospectively reviewed. Personal medical data such as age, comorbidities, preoperative scan, American Society of Anesthesiologists (ASA) score, and operative indication were collected for each patient. Data on the following comorbidities were retrieved: diabetes mellitus, cardiac disease (atrial fibrillation or valvulopathy), hypertension, active smoking, chronic renal failure (defined as glomerular filtration rate <60 ml/min/1.73 m^2^), malnutrition, and obesity (BMI > 30 kg/m^2^). Surgical data of the following were also retrieved: laparoscopic vs. open approach, ultrasound-guided procedure, associated arteriography, stent type, radiation dose and time, and operative time. Thirty-day postoperative morbidity was defined according to the Clavien classification (6). The comprehensive complication index (CCI) is based on the complication grading by Clavien classification and records every complication that occurs after an intervention. The overall morbidity is reflected on a continuous scale from 0 (no complication) to 100 (death) (7). Length of stay was measured from the day of surgery. Six-month primary and secondary stent patencies were also collected. Primary patency was defined as the time between the implantation of the stent and the first event, such as stenosis or occlusion, i.e., the event free time following the operation. Secondary patency was defined as target vessels revascularized after occlusion.

In cases of missing data, patients were reached out for obtaining complete records. Two groups were identified on the basis of the initial clinical presentation: those operated for a GAA and those operated electively for chronic symptoms or DS.

The primary endpoint was to compare 30-day postoperative complications between the two groups. The secondary endpoint was to evaluate the safety of direct stenting during the surgical release with primary and secondary patencies and to compare the operating time and length of stay between the two groups.

Statistical analyses were conducted by using the software package SPSS® version 26 (IBM, Armonk, NY, United States). Mann–Whitney *U* test and Fisher’s exact test were used for independent categorical variables as non-parametric tests. Statistical significance was retained with a double-sided *p*-value <0.05.

The study protocol was in accordance with the Declaration of Helsinki and approved by the local Ethical Committee (Ref. number 2022-00671).

## Results

Overall, 13 patients were treated for MALS in our institution. All had preoperative CT scans done on them, and two also had preoperative ultrasound performed. The patients were treated between 6 days and 14 months after the initial evaluation. Baseline characteristics are detailed in Table 1. Seven patients (54%) were included in the Dumbar syndrome group (DG) and six patients (46%) underwent a semiurgent MALS repair post-embolization for GAA bleeding (BG).

**Table 1:** Demographic characteristics.

Variable	DG (*n* = 7)	BG (*n* = 6)	*p*-value
**Sex, *n* (%)**			
Female	4 (57)	3 (50)	>0.99
Male	3 (43)	3 (50)	>0.99
Median age (IQR), years	59 (27–72)	63 (56–74)	0.63
Median BMI (IQR)	21 (19–33)	26 (23–30)	0.39
**BMI, class, *n* (%)**			
Malnutrition	1 (14)	0 (0)	>0.99
Normal	4 (57)	4 (66)	>0.99
Overweight	0 (0)	1 (17)	0.46
Obese	2 (28)	1 (17)	>0.99
**Coexisting disorders, *n* (%)**			
Diabetes mellitus	1 (14)	0 (0)	>0.99
Hypertension	3 (43)	0 (0)	0.19
Cardiopathy	3 (43)	0 (0)	0.19
Renal disorder	0 (0)	0 (0)	NA
Active smoking	2 (28)	1 (17)	>0.99
**ASA, *n* (%)**			
I–II	4 (57)	6 (100)	0.56
III–IV	3 (43)	0 (0)	0.19

DG, dumbar syndrome group; BG, bleeding group; BMI, body mass index; IQR, interquartile range; NA, not applicable; ASA, American Society of Anesthesiologists.

The four-port laparoscopic approach was performed in almost all patients: there were 12 laparoscopy procedures and 1 median laparotomy. Perioperative ultrasound was used in 11 patients (85%).

Eleven (85%) patients underwent an endovascular CT revascularization. The procedure of left axillary cut down access was performed in seven (64%) patients, whereas percutaneous femoral access was performed in five (45%). CT angioplasty was performed in six (55%) patients and additional stenting was necessary in all (100%). One patient underwent additional SMA stenting. A covered balloon expandable stent was used in most patients (92%). One stent was used per patient on average. The median fluoroscopy time was 20.5 min (IQR: 11.5–26.6), median x-ray dose was 26.05 Gycm^2^ (IQR: 12.5–56.9), and 120 ml (IQR: 74–148) of iodinated contrast media were used. Ten patients were discharged with dual antiplatelet therapy recommended for 3 months, and none were given therapeutic anticoagulation.

In the DG, the median age was 59 years old (IQR: 27–72). Five patients (83) underwent endovascular CT revascularization. The median delay from symptoms to surgical treatment was 3 months (IQR: 3–12) and the median length of stay was 4 days (IQR: 3–5).

In the BG, the median age was 63 years old (IQR: 56–74). All patients (*n* = 6) underwent an associated endovascular CT revascularization. Median treatment delay for the celiac artery compression was 8 days (IQR: 6–41) since admission for GAA rupture. The median length of stay was 16 days (IQR: 8–24).

Patients in the DG who underwent MAL resection had a significantly shorter length of stay (*p* = 0.02). The total operative time was longer in the BG (*p* = 0.06). The median follow-up was 6 months (IQR: 1–12). Two patients in the BG suffered an early major complication (Clavien > 3a) and required reintervention with a higher CCI. One of them underwent a CT stent occlusion and the other CT artery dissection. No case of 30-day mortality was reported. The surgical details and complications are summarized in Table 2.

**Table 2:** Surgical details and postoperative complications.

Variable	DG (*n* = 7)	BG (*n* = 6)	*p*-value
**Surgery, *n* (%)**			
Median laparotomy	0 (0)	1 (17)	0.46
Laparoscopy	7 (100)	5 (83)	0.46
Intraoperative ultrasound	6 (86)	5 (83)	>0.99
**Endovascular procedure, *n* (%)**			
CTA stent	5 (71)	6 (100)	0.46
Non-covered self-expandable stent	1 (14)	0 (0)	>0.99
Covered balloon expandable stent	4 (57)	6 (100)	0.56
Median length (IQR), mm	29 (23–35)	23 (19–29)	0.39
Median diameter (IQR), mm	6 (6–8.5)	8 (7.5–8.3)	0.32
Median time fluoroscopy (IQR), min	11.25 (10–38)	20.5 (14–25)	0.25
Median x-ray dose (IQR), Gycm^2^	26.8 (10–84)	26 (11–57)	0.90
Median iodinated contrast used (IQR), ml	115 (99–144)	120 (45–154)	0.90
**Complications, *n* (%)**			
Any complication	1 (14)	4 (66)	0.10
Clavien > 3a	0 (0)	2 (33)	0.19
Median CCI (IQR)	20.9 (20.9–20.9)	28.15 (21.33–58.10))	NA
Median duration (IQR), min	126 (97–233)	234 (205–258)	0.07
Median lengths of stay (IQR), days	4 (3–5)	16 (8–24)	0.02

DG, Dumbar syndrome group; BG, bleeding group; CTA, celiac trunk artery; NA, not applicable; IQR, interquartile range; CCI, comprehensive complication index.

At 6 months, the primary and secondary CT stent patencies were 82% and 100%, respectively. There were no high-flow GAA recurrences.

## Discussion

This cases series shows that MAL resection associated with stenting seems to be a safe procedure in trained hands for patients with DS. Laparoscopy release of the MAL is now the standard procedure (8, 9) but may require a conversion in case of a massive supraceliac bleeding. The only patient operated with a median laparotomy required a duodenal switch in the context of complications that arose from gastroduodenal bleeding following endoscopy, which was associated with celiac hypertension caused by the MAL. MAL release was performed during the same procedure and this is the reason why open access was preferred. In all other patients, a laparoscopic approach was followed with no conversion. In the literature, a conversion rate of 9.1% was reported by Jimenez et al. on a series review of 400 patients between 1963 and 2012 in the English language (10). This high rate may be related to the learning curve of this rare procedure. The difference between the conversion rate in the literature and that in the present study could be explained by the fact that all patients were operated upon by the same hepatobiliary team in our cohort. To our knowledge, no such standardized procedure has been described to date.

We observed that the operative time and postoperative complications were lower in the DG than in the BG. This could be explained by increased difficulties during surgery in those in the BG because of the presence of a retroperitoneal hematoma or hemoperitoneum.

Two patients in the BG had major complications (Clavien > 3a) and needed reintervention. These complications were related to the stents. Specifically, one patient underwent a stent occlusion on postoperative day (POD) 2. The patient was asymptomatic and the stent thrombosis was diagnosed by using routine duplex ultrasound. We decided to perform a thrombo-aspiration procedure using AngioJet (Boston Scientific, Natick, MA, United States). Proximal stent extension, with a covered balloon expandable stent, was performed through a percutaneous left femoral access. The follow-up was uneventful and the patient was discharged on POD 9.

The second patient underwent a CT dissection due to stent displacement with distal stent occlusion. This patient was also asymptomatic, and the aortic dissection was diagnosed by performing a CT-scan. Dissection stenting was performed on POD 5 using a covered balloon expandable stent. Because of the complexity of the first CT artery catheterization procedure, a left axillary cut down was preferred in this case.

There is no clear evidence in the literature on whether endovascular revascularization should be performed in all cases. The timing is also not well established. Cienfuegos et al. proposed an algorithm in which endovascular treatment will be provided if a patient presents with recurrence or persistence of symptoms after laparoscopic release (11). Their study included 13 patients with Dunbar syndrome, and all of them were operated by laparoscopy; good early results were achieved with 69.2% free from symptoms. Berard et al. and Columbo et al. also suggested percutaneous transluminal angioplasty (PTA) for patients with recurrence or persistence of symptoms (12, 13). In the series of Columbo et al., 7/21 (33%) patients needed PTA because of persistent symptoms of CT stenosis. One of the largest prospective series was published by Brody et al. They treated 42 patients with a laparoscopic release of the MAL and studied the predictors of clinical outcome regarding CT velocity and SF-36 score. Interestingly, they found that age and CT expiratory velocity were significant independent predictors (14).

**Figure 1:**
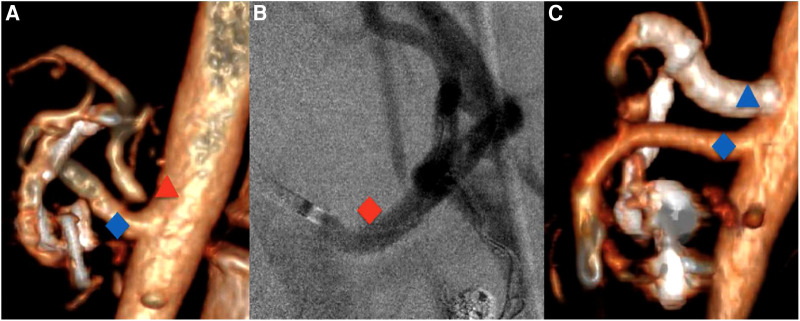
(**A**) Preoperative 3D computed tomography with the CT stenosis (red triangle) and the patent SMA (blue diamond). (**B**) Perioperative CT arteriography after stenting (red diamond). (**C**) Postoperative 3D computed tomography with the patent CT stents (blue triangle) and SMA (blue diamond). CT, celiac trunk; SMA, superior mesenteric artery.

In our center, all patients who underwent MAL release were subsequently managed by a vascular surgeon. A CT angiography is performed after MAL release through femoral or left brachial access (Figure 1). A >70% stenosis was considered significant and was treated by angioplasty and stenting. Interestingly, despite MAL release and intraoperative ultrasound, 11/13 (85%) required additional CT stenting because of persistent stenosis seen on the arteriography.

Although our sample size was small, we could observe a non-significant difference between groups regarding the CCI in favor of patients who underwent elective surgery because of the low incidence of this condition. This patient group had less postoperative complications. The median length of stay is also significantly longer in patients operated in a semi-emergency situation: 16 days vs. 4 days in elective patients (*p* = 0.02).

To our knowledge, this is the first study comparing patients operated for Dunbar syndrome vs. GAA rupture in CT compression.

Statistical analyses are limited by the retrospective design of the study and the sample size. But only a few authors presented a series of more than 10 cases (10–15). With regard to the experience that we acquired in our center, the systematic combined approach of a laparoscopic release of MAL with stenting during the same procedure seems to be a feasible and a safe technique. In the light of these results, we propose that every patient with an incidental finding of an MAL should undergo systematic investigations. In the event of a symptomatic patient presenting with Dunbar syndrome, a possibility for an evaluation of a laparoscopic release of the MAL and stenting of TC or SMA should be examined.

## Conclusion

We report the result of a combined approach of a laparoscopic release of the median arcuate ligament and stenting during the same procedure, and this approach must be systematically discussed and followed in symptomatic patients.

## Data Availability

The original contributions presented in the study are included in the article/Supplementary Material, further inquiries can be directed to the corresponding author.
